# Ginsenoside Rd ameliorates muscle wasting by suppressing the signal transducer and activator of transcription 3 pathway

**DOI:** 10.1002/jcsm.13084

**Published:** 2022-09-20

**Authors:** Yoseph Toni Wijaya, Tania Setiawan, Ita Novita Sari, Keunwan Park, Chan Hee Lee, Kae Won Cho, Yun Kyung Lee, Jae‐Young Lim, Jeong Kyo Yoon, Sae Hwan Lee, Hyog Young Kwon

**Affiliations:** ^1^ Department of Integrated Biomedical Science Soonchunhyang University Cheonan Republic of Korea; ^2^ Soonchunhyang Institute of Medi‐bio Science (SIMS) Soonchunhyang University Cheonan Republic of Korea; ^3^ Natural Product Informatics Research Center Korea Institute of Science and Technology Seoul Republic of Korea; ^4^ Program of Material Science for Medicine and Pharmaceutics, Department of Biomedical Science Hallym University Chuncheon Republic of Korea; ^5^ Institute of Aging Seoul National University Seoul Republic of Korea; ^6^ Department of Rehabilitation Medicine, Seoul National University Bundang Hospital Seoul National University College of Medicine Seongnam Republic of Korea; ^7^ Liver Clinic Soonchunhyang University Cheonan Hospital Cheonan Republic of Korea

**Keywords:** cachexia, ginsenoside Rd, muscle wasting, sarcopenia, signal transducer and activator of transcription 3

## Abstract

**Background:**

The effects of some drugs, aging, cancers, and other diseases can cause muscle wasting. Currently, there are no effective drugs for treating muscle wasting. In this study, the effects of ginsenoside Rd (GRd) on muscle wasting were studied.

**Methods:**

Tumour necrosis factor‐alpha (TNF‐α)/interferon‐gamma (IFN‐γ)‐induced myotube atrophy in mouse C2C12 and human skeletal myoblasts (HSkM) was evaluated based on cell thickness. Atrophy‐related signalling, reactive oxygen species (ROS) level, mitochondrial membrane potential, and mitochondrial number were assessed. GRd (10 mg/kg body weight) was orally administered to aged mice (23–24 months old) and tumour‐bearing (Lewis lung carcinoma [LLC1] or CT26) mice for 5 weeks and 16 days, respectively. Body weight, grip strength, inverted hanging time, and muscle weight were assessed. Histological analysis was also performed to assess the effects of GRd. The evolutionary chemical binding similarity (ECBS) approach, molecular docking, Biacore assay, and signal transducer and activator of transcription (STAT) 3 reporter assay were used to identify targets of GRd.

**Results:**

GRd significantly induced hypertrophy in the C2C12 and HSkM myotubes (average diameter 50.8 ± 2.6% and 49.9% ± 3.7% higher at 100 nM, vs. control, *P* ≤ 0.001). GRd treatment ameliorated aging‐ and cancer‐induced (LLC1 or CT26) muscle atrophy in mice, which was evidenced by significant increases in grip strength, hanging time, muscle mass, and muscle tissue cross‐sectional area (1.3‐fold to 4.6‐fold, vs. vehicle, *P* ≤ 0.05; *P* ≤ 0.01; *P* ≤ 0.001). STAT3 was found to be a possible target of GRd by the ECBS approach and molecular docking assay. Validation of direct interaction between GRd and STAT3 was confirmed through Biacore analysis. GRd also inhibited STAT3 phosphorylation and STAT3 reporter activity, which led to the inhibition of STAT3 nuclear translocation and the suppression of downstream targets of STAT3, such as atrogin‐1, muscle‐specific RING finger protein (MuRF‐1), and myostatin (MSTN) (29.0 ± 11.2% to 84.3 ± 30.5%, vs. vehicle, *P* ≤ 0.05; *P* ≤ 0.01; *P* ≤ 0.001). Additionally, GRd scavenged ROS (91.7 ± 1.4% reduction at 1 nM, vs. vehicle, *P* ≤ 0.001), inhibited TNF‐α‐induced dysregulation of ROS level, and improved mitochondrial integrity (*P* ≤ 0.05; *P* ≤ 0.01; *P* ≤ 0.001).

**Conclusions:**

GRd ameliorates aging‐ and cancer‐induced muscle wasting. Our findings suggest that GRd may be a novel therapeutic agent or adjuvant for reversing muscle wasting.

## Introduction

Myotoxic drugs, aging, cancers, and other diseases, such as chronic kidney disease and heart failure, can cause muscle wasting.^1^ Sarcopenia is a multifactorial syndrome in geriatric patients that is associated with a sedentary lifestyle, oxidative stress, mitochondrial integrity, altered proteostasis, and inflammation.^2^ Cachexia is a wasting syndrome caused by underlying illness and associated with anorexia, inflammation, insulin resistance, and increased muscle protein breakdown.^3^ Cancer cachexia is the term used to refer to muscle atrophy that occurs in cancer patients. It is characterized by a significant increase in the levels of pro‐inflammatory cytokines, such as TNF‐α, IFN‐γ, and interleukin‐6 (IL‐6).^4^ Approximately 80% of cancer patients are affected by cachexia, which leads to a poor prognosis and accounts for 20%–30% of cancer‐associated mortality.^5^ Muscle wasting decreases the quality and duration of life; therefore, it is essential to develop novel therapeutic strategies against it. Currently, there are no effective therapeutics that have a protective effect against muscle atrophy.^5^

Balancing myofibrillar protein synthesis and breakdown is essential in maintaining muscle mass. Several signal transduction pathways, including the AKT/mammalian target of rapamycin (mTOR), unfolded protein response, FoxO, Janus kinase (JAK)/signal transducer and activator of transcription (STAT), and ubiquitin‐proteasome system (UPS) pathways, are involved in muscle hypertrophy and wasting.^6^ Muscle hypertrophy is associated with an increase in muscle mass, and it is the result of an increase in the size of pre‐existing skeletal muscle fibres due to up‐regulated protein synthesis without an apparent variation in myofibre number.^7^ The mTOR pathway is a key driver of protein synthesis; therefore, its inhibition by therapeutic drugs results in muscle atrophy,^8^ which is associated with shrinkage of myofibres and involves a shift toward decreased protein synthesis and increased protein degradation.^9^, ^10^ It is reported that protein degradation is driven by the autophagy‐lysosome pathway, two ubiquitin E3 ligases (muscle atrophy F‐box [atrogin‐1] and muscle RING finger [MuRF‐1]), and the activation of proteasome degradation.^11^ In cancer patients, the IL‐6/JAK/STAT3 pathway is highly up‐regulated and associated with muscle atrophy.^12^ Additionally, JAK/STAT signalling is highly expressed in old mice, and its inhibition leads to the recovery of muscle function.^13^ Furthermore, STAT3 phosphorylation leads to the activation of atrogin‐1, MuRF‐1, and MSTN in cancer patients with cachexia.^11^ Mitochondrial quality and function are impaired during oxidative stress and in cancer. This leads to excessive production of reactive oxygen species (ROS) and contributes to muscle wasting.^14^ STAT3 and ROS are important in muscle‐wasting conditions, which makes them potential targets in the treatment of such conditions.

*Panax ginseng* C. A. Meyer (*Panax ginseng*) has been shown to improve muscle strength and reduce muscle damage in mice.^15^ Therefore, we hypothesized that ginseng‐derived components might have a protective effect against muscle atrophy. Thus, we screened ginseng components and found that GRd has a protective effect against muscle atrophy (*Figure* S1). It has been previously shown that GRd inhibits epidermal growth factor receptor (EGFR) signalling and suppresses cancer stem cell−like properties in colorectal cancer (CRC) cells, which result in the reduction of CRC metastasis.^16^ Similarly, the anti‐tumour activities of GRd in several other cancer types have been reported; however, the effect of GRd on muscle wasting is currently unknown. In this study, we showed that GRd exhibits a muscle anti‐atrophic effect in C2C12 cells and HSkMs. Furthermore, GRd efficiently protected against muscle atrophy in mice with cancer cachexia and aging‐induced atrophy.

## Materials and methods

### Reagents/cell cultures

Ginsenoside Rd^17^ (purity ≥98%) was purchased from Chem Faces (Wuhan, China), and other ginseng‐derived components were obtained from Korean Ginseng Corporation (Daejeon, Korea). Mouse C2C12 myoblast and Lewis lung carcinoma (LLC1) were obtained from ATCC (ATCC® CRL‐1772™ and ATCC® CRL‐1642™, respectively). CT26 and human skeletal muscle (HSkM) were purchased from Korean Cell Line Bank (80009, KCLB) and Gibco™ (A12555, Gibco™), respectively. Other detailed information is provided in Supplementary Materials and Methods.

### Animal study

All animal experiments were approved by the Soonchunhyang University Animal Care and Use Committee and maintained in a pathogen‐free environment with 12 h light–dark cycles and fed with regular normal chow. For the sarcopenia experiment, C57BL/6 male mice (3‐ to 4‐month‐old mice, *n* = 8, control adults) or (23‐ to 24‐month‐old mice, *n* = 16, old) were orally administered either vehicle (0.9% NaCl) or GRd (10 mg/kg BW) daily for 36 days (5 weeks). For the cancer cachexia experiment, LLC1 and CT26 cells (2.0 × 10^6^ cells/mouse) were injected subcutaneously (s.c.) in the right hind flank into male C57BL/6 and BALB/c mice (10‐week‐old), respectively (*n* = 12). When tumours were palpable on day 6, the mice were blindly and randomly divided into two groups and orally administered either vehicle or GRd (10 mg/kg BW) daily for 16 days. Tumour volume was calculated with the formula 0.52*(length*width^2^), and the measurement was performed using a digital calliper. The grip strength was measured every 7 and 3 days for sarcopenia and cancer cachexia, respectively, using the grip strength meter (Ugo Basile, Italy). The hanging test was performed using Kondziela's inverted screen test.

### STAT3 reporter assay

STAT3 reporter assay was described previously^18^ with slight modifications. The plasmid used for STAT3 reporter is pRRL.sin‐18.ppt. STAT3‐GFP.pre (STAT3‐GFP, Addgene plasmid #110495).

### Binding target prediction of GRd

The evolutionary chemical binding similarity (ECBS) method, a machine learning model originally developed to predict chemical binding similarity,^19^ was applied to predict the potential targets of GRd. Among the variants of the ECBS model, the target‐specific ensemble ECBS (TS‐ensECBS) model was used to screen binding targets because of its high accuracy.^19^ A collection of TS‐ensECBS models generated for a total of 7690 targets enabled comprehensive target‐binding predictions for GRd. The predicted target scores were analysed to estimate the potential binding targets of GRd (*Table* S1).

### Biacore assay

Biacore assay was performed with a Biacore 2000 (GE Healthcare, USA); 100 mM NHS and 200 mM EDC were mixed in equal volumes and injected into a closed HC1000M chip (XanTec Bioanalytics) at a rate of 30 μL/min.

### Statistical analysis

All data were performed with two to five independent replicates (as indicated in the figure legends) and the results are presented as mean ± SEM (standard error mean) and analysed by GraphPad Prism software. The experimental groups were compared using one‐way or two‐way analysis of variance (ANOVA) test followed by Tukey's multiple comparisons test, as indicated. The main effects of GRd treatment, time, and an interaction were determined with Mixed‐effects model (REML). The main effects model was used to assess the trajectory of GRd across treatments, while the interaction model was used to assess the differential effect of time based on treatment received, with a focus on the factors of time, treatment received, and the interaction between time and treatment received. The mixed model takes into account the correlation between repeated measures taken within a subject and makes use of all available data on each subject. *P*‐values <0.05 were considered statistically significant.

### Additional methods

Additional details regarding the methods and materials are provided in the Supplementary Materials and Methods.

## Results

### GRd protects C2C12 myotubes against TNF‐α/IFN‐γ (TI)‐induced myotube atrophy

**Figure 1:**
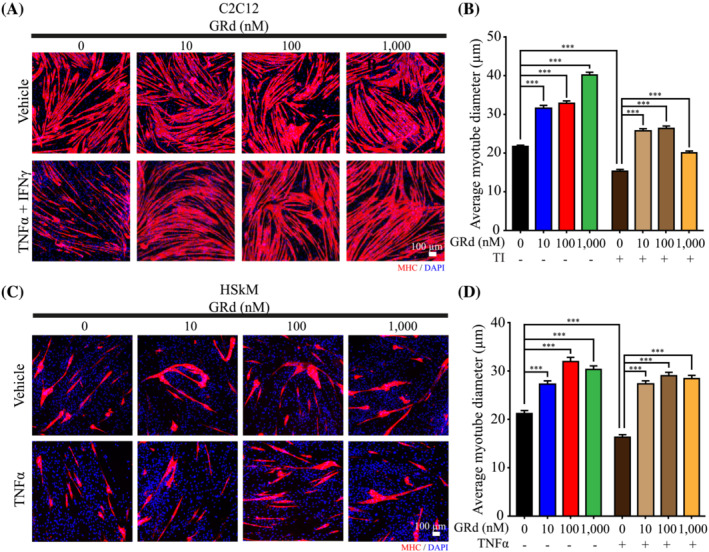
GRd protects C2C12 and HSkM myotubes against muscle cell atrophy. (A, B) C2C12 myotubes were treated with GRd at the indicated concentration in the presence or absence of TI (TNF‐α at 20 ng/mL and IFN‐γ at 100 U/mL) for 24 h. (C, D) HSkM myotubes were treated with GRd at the indicated concentration in the presence or absence of TNF‐α (10 ng/mL) for 24 h. Then, C2C12 and HSkM myotubes were stained with anti‐MHC ab. (A, C) Representative images were shown. (B, D) Average myotube diameter was measured by ImageJ software. The data were shown as mean ± SEM of more than 100 myotubes from 10 randomly chosen fields. One‐way ANOVA (B–D) followed by Tukey's multiple comparisons were used to compare between data (*P ≤ 0.05; **P ≤ 0.01; ***P ≤ 0.001).

We sought to identify ginseng‐derived components that have an in vitro protective effect against TI‐induced muscle wasting. After 4 days of differentiation, C2C12 myotubes were treated with ginseng‐derived components together with TI. As expected, myotubes diameter was decreased following the treatment with TI. Interestingly, GRd had the highest anti‐atrophic effect among the ginseng components tested in TI‐treated C2C12 myotubes, as evidenced by the hypertrophy in GRd‐treated C2C12 myotubes compared to that in the other groups (*Figure* S1A, B). Thus, we focused on GRd and determined its dose–response effect against myotube atrophy. C2C12 myotubes were treated with several concentrations of GRd (10–1000 nM) with or without TI, after which myotube atrophy was determined. The results showed that GRd significantly increased the diameter of C2C12 myotubes in a dose‐dependent manner (*Figure* 1A,B). The decreased diameter of C2C12 myotubes treated with conditioned media obtained from CT26 or LLC1 was consistently increased by GRd (*Figure* S1C, D), which suggests that GRd can protect against pathological muscle wasting.

### GRd ameliorates myotube atrophy in HSkMs

GRd ameliorated atrophy in the mouse C2C12 myotubes; therefore, we evaluated the anti‐atrophic effects of GRd in human myotubes. Myotube atrophy in HSkMs was induced with TNF‐α, after which the effect of GRd on myotube diameter was investigated. TNF‐α induced myotube atrophy, which was evidenced by a smaller myotube diameter (*Figure* 1C,D). Consistent with the results obtained from the tests performed on mouse C2C12 myotubes, it was found that myotube diameter was much larger following treatment with GRd than with TNF‐α alone (*Figure* 1C,D). To confirm this in an independent method, HSkM was treated with HT29‐derived conditioned media. The decreased diameter of HSkM myotubes with HT29‐conditioned media was rescued by GRd (*Figure* S1C,D). These data indicate that GRd ameliorates TNF‐α or HT29‐conditioned media‐induced atrophy in human myotubes.

### GRd improves muscle function and mass in aged mice

**Figure 2:**
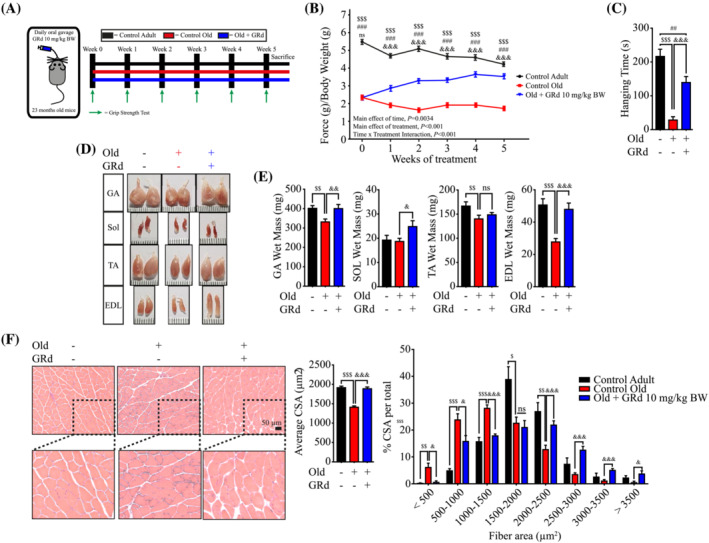
GRd improves muscle function and mass in aged mice. (A) Experiment design of sarcopenia model with GRd treatment. Aged C57BL/6 mice (23 to 24 months old) were orally administrated with GRd (10 mg/kg body weight/day) or vehicle daily for 36 days. Adult C57BL/6 mice (4 months old) were used as healthy control. Four months old mice (*n* = 8), 23 to 24 months old mice + vehicle (*n* = 8), and 23 to 24 months old mice + GRd 10 mg/kg (*n* = 8). (B) Grip strength was evaluated every 7 days (the main effect of treatment, *P* < 0.001; the main effect of time, *P* = 0.0034; interaction, *P* < 0.001). (C) Hanging test was performed at day 34. (D, E) Skeletal muscles, including GA, SOL, TA, and EDL, were dissected at day 36. Representative images of skeletal muscles were shown. The weights of skeletal muscles and organs were determined. (F) GA muscles were stained with H&E staining, and representative images were shown. The average cross‐sectional area (CSA) of GA muscle fibre was quantified by ImageJ and plotted depending on the frequency as indicated. The data were shown as mean ± SEM. One‐way (C, E, F) or two‐way (B, F) ANOVA followed by Tukey's multiple comparisons were used to compare between data (ns, not significant; ^$^ *P* < 0.05, ^$$^ *P* < 0.01, ^$$$^ *P* ≤ 0.001, control adult vs. control old; ^##^ *P* < 0.01, ^###^ *P* ≤ 0.001, control adult vs. old + GRd; ^&^ *P* < 0.05, ^&&^ *P* < 0.01, ^&&&^ *P* ≤ 0.001, control old vs. old + GRd).

Based on the observed anti‐atrophic effect of GRd on myotubes *in vitro*, we investigated whether GRd protects against muscle wasting *in vivo*. The effects of GRd on muscle wasting were assessed in aged mice (23–24 months old) and control adult mice (4 months old). The animals were treated with vehicle or GRd daily for 5 weeks (*Figure* 2A). As expected, the aged mice exhibited lower grip strength than the control adult mice did; however, GRd significantly improved grip strength (*Figure* 2B; the main effect of GRd treatment, *P* < 0.001), although body weight was not affected (*Figure* S2A). The grip strength of GRd treatment increased over time (*Figure* 2B; main effect of time, *P* = 0.0034). To further corroborate the results of the grip strength test, a hanging test was performed using Kondziela's inverted screen. Hanging time was shorter among the aged mice than among the control mice but was significantly improved by GRd (*Figure* 2C). Interestingly, the weights of the gastrocnemius (GA), soleus (SOL), and extensor digitorum longus (EDL) muscles were significantly higher in the GRd‐treated group than in the control old mice. However, there was no difference in the weight of the tibialis anterior (TA) muscle between the groups (*Figure* 2D,E). Furthermore, liver, spleen, and lung weights were not different between the vehicle‐ and GRd‐treated mice; however, heart weight was significantly lower in the GRd‐treated mice than in the control old mice (*Figure* 2B). To evaluate differences in muscle tissue between groups, haematoxylin and eosin (H&E) staining was carried out, after which the cross‐sectional area (CSA) of the GA muscle fibre was determined. The GA muscle CSA was much smaller in the aged group than in the control group (*Figure* 2F); however, it was significantly higher in the GRd‐treated group than in the vehicle‐treated group. The average CSA in the GRd‐treated group was 33.83% higher than that in the vehicle‐treated group but similar to that in the control group (*Figure* 2F). These results indicate that GRd can improve muscle mass and function in aged mice.

### GRd ameliorates Lewis lung carcinoma (LLC1)‐ and CT26‐induced cancer cachexia *in vivo*

**Figure 3:**
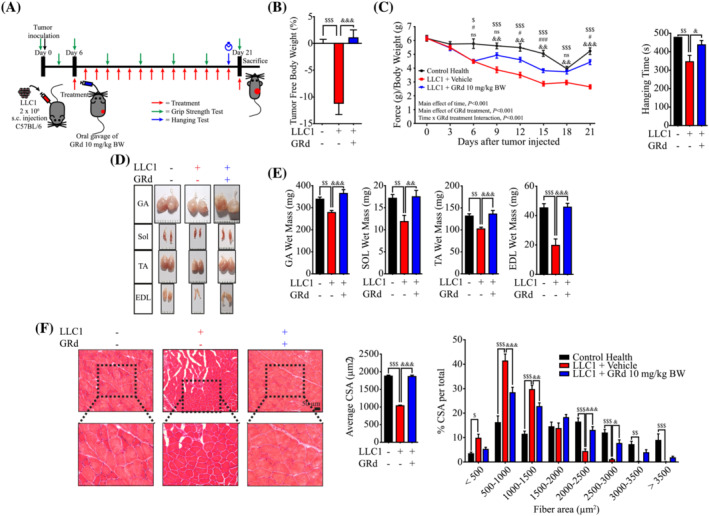
GRd ameliorates Lewis lung carcinoma (LLC1)‐induced cancer cachexia *in vivo*. (A) Experiment design of LLC1‐induced cancer cachexia. C57BL/6 mice were injected subcutaneously (s.c.) with LLC1 cells. Then, after 6 days of injection, mice were orally administrated with GRd (10 mg/kg body weight/day) or vehicle daily for 16 days. Mice without tumour inoculation was used as control health. Control (*n* = 6), LLC1 + vehicle (*n* = 6), and LLC1 + GRd 10 mg/kg (*n* = 6). (B) Tumor‐free body weight was measured in the day 21. (C) Muscle performance for grip strength was evaluated every 3 days (the main effect of treatment, *P* < 0.001; the main effect of time, *P* < 0.001; interaction, *P* < 0.001), and hanging test was conducted on day 20. (D, E) Skeletal muscles, including GA, SOL, TA, and EDL, were dissected after 21 days, and the weights were determined. Representative images of skeletal muscles were shown. (F) GA muscles were stained with H&E staining, and representative images were shown. The average cross‐sectional area (CSA) of GA muscle fibre was quantified by ImageJ and plotted depending on the frequency as indicated. The data were shown as mean ± SEM. One‐way (B, C, E, F) or two‐way (C, F) ANOVA followed by Tukey's multiple comparisons were used to compare between data (ns, not significant; ^$^ *P* ≤ 0.05, ^$$^ *P* ≤ 0.01,^$$$^ *P* ≤ 0.001, control health vs. LLC1 + vehicle; ^#^ *P* ≤ 0.05, ^###^ *P* ≤ 0.001, control health vs. LLC1 + GRd; ^&^ *P* ≤ 0.05, ^&&^ *P* ≤ 0.01, ^&&&^ *P* ≤ 0.001, LLC1 + vehicle vs. LLC1 + GRd).

Cancer‐induced cachexia is characterized by loss of skeletal muscle in patients; therefore, we determined whether GRd can protect against muscle loss in a mouse model of LLC1‐induced cancer cachexia. LLC1 cells were subcutaneously injected into the flanks of C57BL/6 mice, after which the mice were orally administered GRd daily for 16 days starting from day 6 when the tumours were palpable. Grip strength was determined every 3 days during the treatment period, and hanging ability was evaluated 1 day before the animals were sacrificed (*Figure* 3A). Decreases in body weight and muscle weight as well as low grip strength and short hanging time were indicative of the successful establishment of LLC1‐induced cancer cachexia in tumour‐bearing mice (*Figures* 3B,C and S3A). Tumour sizes were smaller in GRd‐treated mice than in control mice at the tested concentrations, which indicated the anti‐tumour effect of GRd (*Figure* S3B). Interestingly, GRd noticeably ameliorated body wasting, muscle atrophy, weakness, and loss of strength, which are features of cancer cachexia (*Figures* 3B–E and S3A). Body weight was 11.28% lower in the vehicle‐treated tumour‐bearing group (LLC1 + vehicle) than in the healthy control group. However, it was found that GRd (LLC1 + GRd) ameliorated body weight loss (*Figure* 3B). Moreover, grip strength progressively decreased over time in the vehicle‐treated tumour‐bearing group but was significantly improved by GRd from day 6 (*Figure* 3C; main effect of GRd treatment, *P* < 0.001). Similarly, hanging time was partially improved by GRd (*Figure* 3C). LLC1‐induced decreases in GA, SOL, TA, and EDL muscle weights were also prevented by GRd (*Figure* 3D,E). Heart, lung, kidney, liver, and spleen weights were not different between the vehicle‐ and GRd‐treated groups (*Figure* S3C). H&E staining was performed on GA muscles excised from the treated mice. It was found that, compared to the healthy control mice, the vehicle‐treated tumour‐bearing mice had smaller muscle fibres with smaller average CSAs (*Figure* 3F). However, GRd protected myofibres from the decrease in CSA (*Figure* 3F).

**Figure 4:**
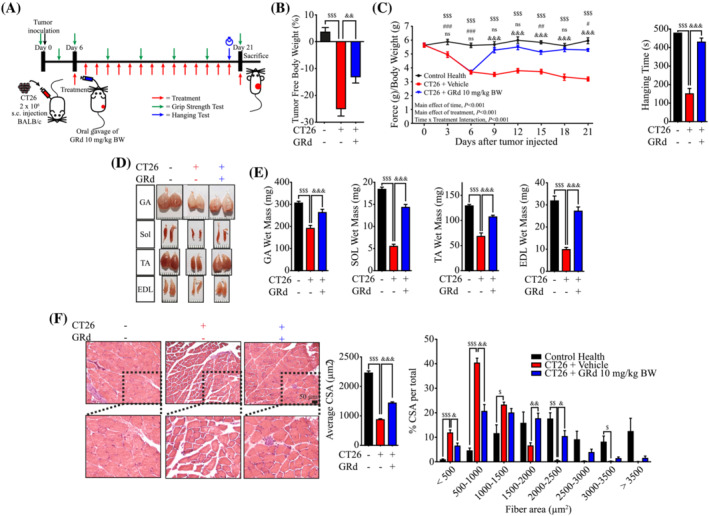
GRd ameliorates (CT26)‐induced cancer cachexia *in vivo.* (A) Experiment design of CT26‐induced cancer cachexia. BALB/c mice were injected subcutaneously with CT26 cells. Then, after 6 days of injection, mice were orally administrated with GRd (10 mg/kg body weight/day) or vehicle daily for 16 days. Mice without tumour inoculation was used as control health. Control (*n* = 6), CT26 + vehicle (*n* = 6), and CT26 + GRd 10 mg/kg (*n* = 6). (B) Tumor free body weight was measured in the day 21. (C) Muscle performance for grip strength was evaluated every 3 days (the main effect of treatment, *P* < 0.001; the main effect of time, *P* < 0.001; interaction, *P* < 0.001), and hanging test was conducted on day 20. (D, E) Skeletal muscles, including GA, SOL, TA, and EDL were dissected after 21 days, and the weights were determined. Representative images of skeletal muscles were shown. (F) GA muscles were stained with H&E staining, and representative images were shown. The average cross‐sectional area (CSA) of GA muscle fibre was quantified by ImageJ and plotted depending on the frequency as indicated. The data were shown as mean ± SEM. One‐way (B, C, E, F) or two‐way (C, F) ANOVA followed by Tukey's multiple comparisons were used to compare between data (ns, not significant; ^$^ *P* ≤ 0.05, ^$$^ *P* ≤ 0.01, ^$$$^ *P* ≤ 0.001, control health vs. CT26 + vehicle. ^#^ *P* ≤ 0.05, ^##^ *P* ≤ 0.01, ^###^ *P* ≤ 0.001, control health vs. CT26 + GRd. ^&^ *P* ≤ 0.05, ^&&^ *P* ≤ 0.01, ^&&&^ *P* ≤ 0.001, CT26 + vehicle vs. CT26 + GRd).

A mouse model of CT26 CRC‐induced cachexia was also used in the study to validate the therapeutic efficacy of GRd in different cancer cachexia models. BALB/c mice were subcutaneously injected with CT26 cells and administered GRd or vehicle (*Figure* 4A). The results were similar to those obtained for the other cancer cachexia model. Several features of CT26 cancer‐induced cachexia, such as loss of grip strength, weakness, and muscle atrophy, were partially ameliorated by GRd (*Figures* 4B–F and S3D–F; main effect of GRd treatment, *P* < 0.001). Collectively, these results provide evidence that GRd can prevent cancer‐induced muscle wasting *in vivo*.

### GRd interacts with STAT3

**Figure 5:**
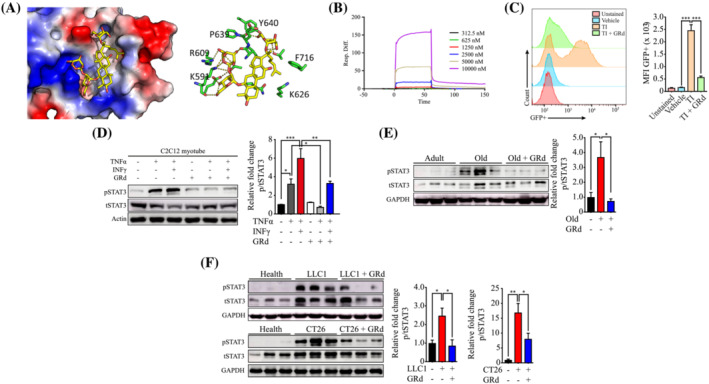
GRd inhibits muscle wasting through direct binding with STAT3. (A) Molecular docking between GRd and STAT3. (B) Biacore analysis was used to analyse the binding ability of GRd to STAT3. (C) C2C12 cells were transduced with STAT3 reporter, and then treated for 8 h with TNF‐α at 20 ng/mL and IFN‐γ at 100 U/mL (TI) in the presence or absence of GRd (100 nM). STAT3 reporter expressions were measured by flow cytometry. (D) C2C12 myotubes were treated for 24 h with TNF‐α at 20 ng/mL and IFN‐γ at 100 u/mL in the presence or absence of GRd (100 nM) as indicated (*n* = 4). Then, the protein levels of pSTAT3/tSTAT3 were evaluated by western blot. Representative images were shown and images were measured by ImageJ software. The data were shown as mean ± SEM. (E, F) GA tissue lysates were isolated from aged mice (*n* = 3), LLC1 (*n* = 6), or CT26‐implanted mice (*n* = 6). Then, the protein levels of pSTAT3/tSTAT3 were evaluated by western blot. Representative images were shown and images were measured by ImageJ software. The data were shown as mean ± SEM. One‐way (C‐F) ANOVA followed by Tukey's multiple comparisons were used to compare between data (*P ≤ 0.05; **P ≤ 0.01; ***P ≤ 0.001).

An evolutionary chemical binding similarity (ECBS) approach, which was previously developed based on a classification similarity‐learning framework, was used to identify the molecular targets of GRd.^19^ The binding targets of GRd were predicted using a collection of target‐specific ensemble models generated for 7690 targets.^20^ The predicted binding scores for each target were used to prioritize potential binding target candidates (Table S1). STAT3 was found to be one of the top‐ranked candidates. It is of particular interest as it is an important mediator of muscle wasting and is considered a drug target in muscle atrophy treatment. To independently validate the ECBS findings, we conducted a molecular docking study to investigate the interaction between GRd and STAT3. The results suggested that GRd had favourable molecular interactions with STAT3 at its binding sites. The predicted binding energy was found to be −8.1 kcal/mol (*Figure* 5A). In the docking model, the sugar moiety in GRd primarily interacted with negatively charged residues of STAT3, such as Lys591 and Arg609, whereas the steroid backbone had hydrophobic interactions with Phe716, Tyr640, and Lys626 (*Figure* 5A). Direct binding between GRd and STAT3 was experimentally confirmed through Biacore analysis, in which STAT3 was immobilized on a chip and GRd was injected to determine its affinity for STAT3. The results indicated that GRd was bound to STAT3 with a dissociation constant (Kd) of 36.3 μM (*Figure* 5B). A STAT3 reporter assay was also performed to verify the interaction between GRd and STAT3. In the assay, the level of green fluorescent protein (GFP), which is a surrogate marker of STAT3, depends on STAT3 transcriptional activity since the reporter has four STAT3 binding sites.^18^ C2C12 cells were transduced with a lentiviral STAT3 reporter and then treated with TI in the presence or absence of GRd (*Figure* 5C). As expected, the GFP level was 14.22‐fold higher following treatment with TI than it was following the control treatment; however, it was significantly reduced by GRd (*Figure* 5C), suggesting that GRd regulates STAT3 activity.

### GRd regulates the STAT3 pathway by suppressing the phosphorylation and nuclear localization of STAT3

**Figure 6:**
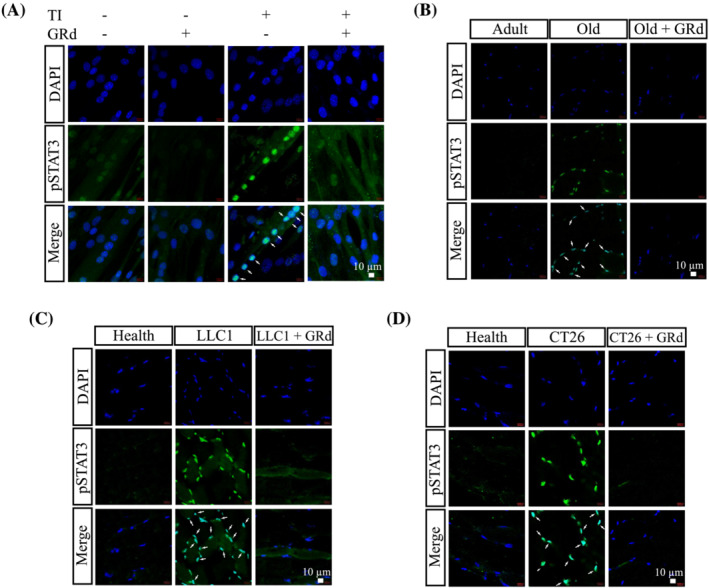
GRd inhibits STAT3 nuclear translocalization. (A) C2C12 myotubes were incubated for 30 min with TI **±** GRd (100 nM) as indicated and stained with antibodies against pSTAT3 (green) and counterstained with DAPI (blue) to determine the localization of STAT3 in the nucleus. (B–D) Cryosections of GA muscles from aged mice, LLC1, and CT26‐implanted mice were stained with antibodies against pSTAT3 (green) and counterstained with DAPI (blue). Representative images were shown. Arrows indicate nuclear localization of STAT3.

Active STAT3 is phosphorylated during muscle wasting^21^, ^22^, ^23^, ^24^; therefore, we investigated whether GRd changes the level of phosphorylated STAT3 *in vitro*. Interestingly, TNF‐α‐ or TI‐induced up‐regulation of STAT3 phosphorylation was significantly reduced by GRd (*Figure* 5D). In a further study, we investigated the lower levels of pSTAT3 *in vivo* by analysing muscle samples obtained from aged mice and cancer (LLC1 or CT26)‐bearing mice. Consistently, the pSTAT3 level was much higher in these mice than in the control mice but was significantly reduced by GRd (*Figure* 5E,F). STAT3 nuclear translocation was also evaluated since activated STAT3 is localized within nuclei.^23^, ^24^ C2C12 cells were treated with TI in the absence or presence of GRd, after which STAT3 localization was determined using a confocal microscope. It was found that STAT3 level was highly up‐regulated and that STAT3 was localized within the nucleus following treatment with TI; however, these effects were inhibited by GRd (*Figure* 6A). Our findings were also confirmed on muscle tissues excised from aged mice and cancer‐bearing mice (*Figure* 6B–D). Taken together, the current findings suggest that GRd binds to STAT3, resulting in the suppression of STAT3 phosphorylation and nuclear translocation.

**Figure 7:**
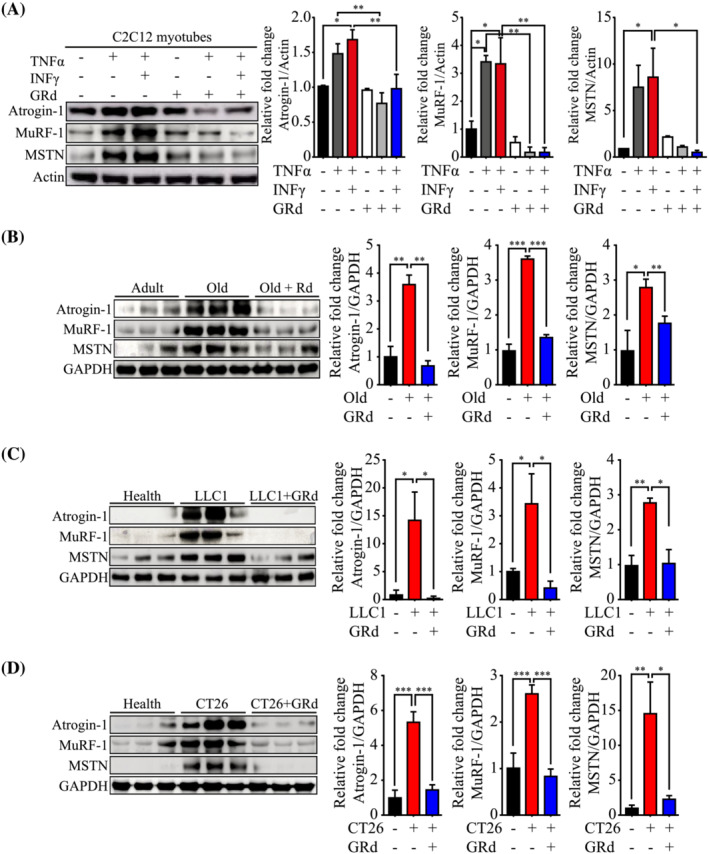
GRd suppresses the downstream pathways of STAT3. (A) C2C12 myotubes were treated for 24 h with TNF‐α at 20 ng/mL, IFN‐γ at 100 U/mL or GRd (100 nM) as indicated, and then protein levels of muscle atrophy‐related genes were evaluated by western blot. The data were shown as mean ± SEM of three to four independent experiments. (B–D) GA tissue lysates were isolated from aged mice‐, LLC1‐, and CT26‐implanted mice, respectively. Then, the protein levels of muscle atrophy‐related genes were evaluated by western blot. Representative images were shown, and images were measured by ImageJ software. The data were shown as mean ± SEM (*n* = 6). One‐way (A–D) ANOVA followed by Tukey's multiple comparisons were used to compare between data (*P ≤ 0.05; **P ≤ 0.01; ***P ≤ 0.001).

MSTN and muscle‐specific E3 ubiquitin ligases, MuRF‐1 and atrogin‐1, are central mediators of muscle wasting and their expression is induced following STAT3 activation.^25^ Thus, we hypothesized that these molecules might be affected by GRd. To test this, C2C12 cells were treated with TNF‐α alone or with the TI combination in the absence or presence of GRd. The levels of MuRF‐1, atrogin‐1, and MSTN were then determined via immunoblotting. As expected, the levels of all three proteins were increased by TNF‐α alone or TI but significantly reduced by GRd (*Figure* 7A). To verify these changes in animal models, muscle‐derived protein lysates obtained from aged mice and cancer (LLC1 or CT26)‐bearing mice were analyzed. Consistently, GRd significantly inhibited the expression of MuRF‐1, atrogin‐1, and MSTN in these groups of mice (*Figure* 7B–D).

### GRd reduces ROS levels and protects mitochondrial integrity

**Figure 8:**
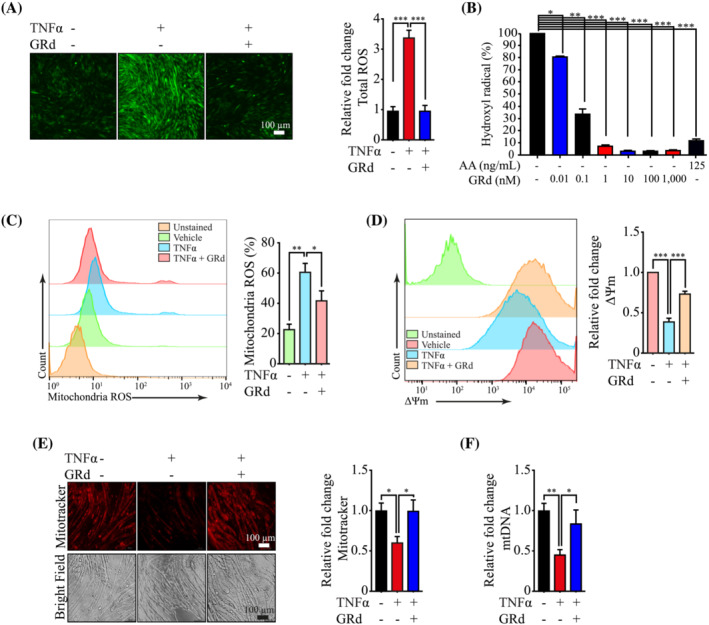
GRd reduces ROS levels and protects mitochondrial integrity. (A) C2C12 myotubes were incubated for 4 h with TNF‐α (20 ng/mL), with/without GRd (100 nM) as indicated, and then analysed using fluorescence microscope to analyse ROS levels. Representative fluorescence images were shown, and fluorescence intensity was quantified using the ImageJ software. The data were presented as mean ± SEM of at least 10 randomly chosen fields of each condition. (B) The effect of GT on scavenging hydroxyl radicals was analysed using iron (II)‐dependent TBA reactive substance. Ascorbic acid (AA) at 125 ng/mL was used as a positive control. Data were shown as mean ± SEM of three independent experiments. (C) C2C12 myoblast was incubated for 24 h with TNF‐α (20 ng/mL) ± GRd (100 nM) as indicated, and mitochondria ROS were measured by flow cytometry. Representative FACS profiles were shown, and the data were presented as mean ± SEM of three independent experiments. (D) C2C12 myoblast was incubated for 24 h with TNF‐α (20 ng/mL) with/without GRd (100 nM) as indicated, and mitochondrial membrane potential (ΔѰm) were measured by flow cytometry. Representative FACS profiles were shown, and the data were presented as mean ± SEM of three independent experiments. (E) C2C12 myoblast was incubated for 24 h with TNF‐α (20 ng/mL) or GRd (100 nM) as indicated, and then the number of mitochondria were measured by fluorescence microscope. Representative fluorescence images were shown, and fluorescence intensity was quantified using the ImageJ software. The data were presented as mean ± SEM of three independent experiment. (F) C2C12 myoblast were differentiated for 72 h with TNF‐α (20 ng/mL) in the presence or absence of GRd (100 nM), and then the mtDNA copy number were quantified by RT‐PCR. The data were presented as mean ± SEM of four independent experiments. One‐way (A–F) ANOVA followed by Tukey's multiple comparisons were used to compare between data (*P ≤ 0.05; **P ≤ 0.01; ***P ≤ 0.001).

STAT3‐induced ROS accumulation in muscle wasting was observed and linked to compromised mitochondrial integrity.^13^ Thus, we hypothesized that GRd may reduce ROS levels by suppressing STAT3 activation and MSTN expression. To test this, we evaluated GRd‐induced changes in total ROS level in C2C12 cells by flow cytometry and fluorescence microscopy. As expected, cellular ROS level was increased by TNF‐α but significantly reduced by GRd (*Figures* 8A and S4). Interestingly, GRd had a strong ROS scavenging activity. The results showed that GRd decreased the levels of free radicals by 92.72% even at a concentration of 1 nM (*Figure* 8B). Thus, we also determined the effect of GRd on mitochondrial ROS levels using the MitoSox™ Red Mitochondrial Superoxide Indicator. Mitochondrial ROS level was also decreased by GRd, which was consistent with the effect of GRd on total ROS level (*Figure* 8C). Higher levels of ROS are associated with a reduction in mitochondrial function, which is indicated by a decrease in mitochondrial membrane potential (ΔΨm).^26^ Thus, we assessed ΔΨm and mitochondrial number using MitoProbe DilC_1_(5) assay kit and MitoTracker™ Orange, respectively. The results showed that ΔΨm and mitochondrial number in C2C12 cells decreased following treatment with TNF‐α; however, this was partially inhibited by GRd (*Figure* 8D,E). Additionally, mitochondrial DNA (mtDNA) copy number, an indicator of mitochondrial biogenesis level,^10^ was decreased by TNF‐α but partially increased by GRd (*Figure* 8F). These results indicate that GRd reduces ROS level and protects mitochondrial integrity via ROS scavenging and suppression of STAT3 signalling, thereby conferring protection against muscle wasting.

## Discussion

Muscle atrophy is associated with various conditions such as cancers, aging, and chronic diseases, including diabetes, chronic kidney diseases, and chronic obstructive pulmonary disease.^1^ It has major negative effects on the quality of life and contributes to a high rate of mortality.^5^ In this study, we showed that GRd has the potential to alleviate muscle wasting in pathophysiological conditions such as cancer and sarcopenia. GRd exerted strong anti‐atrophic effects in both C2C12 and HSkM myotubes. Furthermore, GRd treatment protects against cancer‐induced (LLC1 and CT26) and aging‐induced muscle atrophy following oral administration to mice, which was evidenced by increases in grip strength and CSA of muscle fibres. The results showed that, mechanistically, GRd binds to STAT3 and reduces its phosphorylation, thereby suppressing the expression of several atrophy‐inducing factors such as MSTN, MuRF‐1, and atrogin‐1. Therefore, the STAT3 pathway is a major pathway that GRd suppresses to protect against muscle atrophy.

IL‐6/JAK/STAT3 signalling plays an important role in muscle wasting by regulating inflammatory responses.^27^ It is reported that STAT3 phosphorylation results in an increase in MSTN, atrogin‐1, and MuRF‐1 expression through the activation of the CCAAT/enhancer‐binding protein delta.^25^ Moreover, genetic ablation of STAT3 in a mouse model results in partial amelioration of muscle wasting under the induction of diabetes, chronic kidney disease, and cachexia.^25^ Similarly, in a previous study, pharmacological inhibition of STAT3 with STAT3 inhibitors, such as AG490 (JAK2 inhibitor) and sunitinib, led to the amelioration of muscle wasting in several muscle wasting models.^28^ In the present study, an ECBS approach and molecular docking analysis were used to predict that STAT3 is a target of GRd. Then, we performed Biacore analysis and experimentally confirmed the interaction between STAT3 and GRd. The results showed that, mechanistically, GRd binds to STAT3 and reduces its phosphorylation, thereby suppressing the expression of several atrophy‐inducing factors such as MSTN, MuRF‐1, and atrogin‐1. Therefore, the STAT3 pathway is a major pathway that GRd suppresses to protect against muscle atrophy. Further, the results showed that aging‐ and cancer‐induced muscle atrophy was effectively suppressed by GRd through the suppression of STAT3 phosphorylation and nuclear translocation.

It was also shown that IL‐6 binding to its receptor induces STAT3 phosphorylation, which further leads to skeletal muscle proteolysis and muscle wasting.^29^ Muscle wasting factors, such as MSTN, ROS, activin, and growth differentiation factor 15, as well as inflammatory cytokines, such as TNF‐α, IFN‐γ, and IL‐6, promote consistent STAT3 activation, catabolism by activating UPS, and the autophagy‐lysosome system.^30^ Further, the increased circulatory proinflammatory cytokines IL‐6, TNF‐α, and TGF‐β are associated with decreased mitochondrial biogenesis and function, and an imbalance of mitochondrial dynamics.^31^ TNF‐α and H_2_O_2_ are potent inducers of MSTN, which triggers ROS generation. Thus, MSTN and TNF‐α form a feed‐forward loop that induces muscle wasting through proteasomal‐mediated catabolism.^32^ In the present study, it was found that GRd scavenges ROS and inhibits STAT3 phosphorylation, which results in a decrease in ROS level and down‐regulation of MSTN expression. Further, ROS‐induced decreases in ΔΨm, mitochondrial number, and mtDNA copy number were significantly inhibited by GRd, suggesting that GRd is a potent inhibitor of proteasomal catabolism via the STAT3 pathway. Thus, we propose for the first time that GRd binds to STAT3, reducing the muscle wasting activity of STAT3 by binding to it. However, it is also plausible that GRd might exert its activity through other mechanisms, besides STAT3. Recently, Tang *et al*. demonstrated that GRd treatment enhanced AMP‐activated protein kinase (AMPK)/Sirtuin 1 (SIRT1) interaction, leading to the reduction of oxidative stress, mitochondrial dysfunction, and apoptosis in diabetic retinopathy condition.^33^ As SIRT1 was shown to prevent muscle dysfunction,^34^ it could be possible that the anti‐atrophic effects of GRd might be associated with SIRT1 activation, which warrants further study.

In the Orient, particularly in Korea and China, ginseng roots and root extracts have been traditionally used for many centuries as medicine to revitalize the body and mind, prevent aging, and increase vigour. The major active components of *Panax ginseng* are ginsenosides, polysaccharides, volatile oils, glycopeptides, amino acids, and vitamins. There are major ginsenosides (*Rb1*, *Rb2*, *Rc*, *Rd*, *Re*, and *Rg1*) which comprise more than 80% of the total ginsenosides, and minor ginsenosides (*F1*, *F2*, *Rg3*, *Rh1*, *Rh2*, *compound Y*, *compound Mc*, and *compound K*) that are present at a low concentration in ginseng.^35^ GRd has been shown to have anti‐cancer effects against gastric cancer.^36^ Additionally, by counteracting the effects of miR‐18a and down‐regulating Smad2 expression, GRd suppresses the metastasis of breast cancer.^37^ Recently, it was reported that GRd inhibits the metastasis of CRC cells to the liver, lungs, and kidneys in mice.^16^ Consistently, it was found in the present study that GRd reduced tumour volumes in the mice with LLC1‐ and CT26‐induced cachexia. Together with the findings that GRd could protect C2C12 and HSkM against myotube atrophy, we rationalize that the mechanisms by which GRd exerts its effect in cancer‐induced cachexia might be associated with dual effects, including reducing cancer burden and protecting against muscle atrophy, which warrants further studies. Several ginsenosides (Rb1 and Rb2) show protective effects against muscle wasting via the AKT signalling pathway.^38^ However, the effects of GRd on TI‐induced cellular atrophy were better than those of other ginseng components in our studies. GRd has already been clinically used to treat acute ischaemic stroke and has been shown to have fewer side effects compared to glucocorticoids, indicating that it can safely improve patient outcomes following ischaemic stroke.^39^ Thus, we propose that GRd should be clinically tested for its effect against muscle atrophy.

## Conclusions

In this study, we found that GRd protects against aging‐ and cancer‐induced skeletal muscle wasting by binding to STAT3, enhancing muscle function, and suppressing the expression of protein degradation factors such as MSTN, atrogin‐1, and MuRF‐1. Additionally, GRd reduces ROS levels and protects mitochondrial integrity (*Figure* S5). Taken together, our findings show that GRd may be a suitable therapeutic agent for suppressing muscle atrophy under various pathophysiological conditions.

## Conflicts of interests

The authors declare that they have no competing interests.

## Supporting information


**Table S1.** Predicted binding targets of GRd by ECBS
Figure S1. Ginseng‐derived components protect from TNF‐α/IFN‐γ or conditioned media‐induced muscle cell atrophy.
(A, B) Differentiated C2C12 myotubes were treated with various ginseng‐derived components in the presence or absence of TNF‐α (20 ng/mL) and IFN‐γ (100 U/mL) for 24 h and stained with anti‐MHC Ab. (A) Representative images were shown. (B) Average myotube diameter was measured by ImageJ software. (C, D) Differentiated C2C12 or HSKM myotubes were treated with conditioned media obtained from CT26, LLC1, or HT29, as indicated, for 24 h and stained with anti‐MHC Ab. (C) Representative images were shown. (D) Average myotube diameter was measured by ImageJ software. The data were shown as mean ± SEM of more than 100 myotubes from 10 randomly chosen fields. One‐way ANOVA followed by Tukey's multiple comparisons were used to compare between data (*P ≤ 0.05; **P ≤ 0.01; ***P ≤ 0.001).
Figure S2. GRd maintained organ weights in an aged mouse model.
(A) Body weights were monitored every 7 days. The main effect of treatment, *P* = 0.0249; the main effect of time, *P* < 0.001; interaction, *P* < 0.001. (B) Organs, such as heart, lungs, spleen, and livers were dissected at day 36. The weights of organs were determined. The data were shown as mean ± SEM. Two‐way (A) or one‐way (B) ANOVA followed by Tukey's multiple comparisons were used to compare between data. ns, not significant; ^$^ *P* ≤ 0.05, ^$$^ *P* ≤ 0.01, control adult vs. control old. ^#^ *P* ≤ 0.05, control adult vs. old+GRd; ^&^ *P* < 0.05, control old vs. old+GRd.
Figure S3. GRd maintained body weights and organ weights in cachexia mouse models.
(A‐C) C57BL/6 mice were injected subcutaneously (s.c.) with LLC1 cells. Then, after 6 days of injection, mice were orally administrated with GRd (10 mg/kg body weight/day) or vehicle daily for 16 days. Mice without tumour inoculation were used as control health. Control (*n* = 6), LLC1 + vehicle (n = 6), and LLC1 + GRd 10 mg/kg (n = 6). Body weights (the main effect of treatment, *P* = 0.0902; the main effect of time, *P* < 0.001; interaction, *P* < 0.001) and tumour volumes (the main effect of treatment, *P* < 0.001; the main effect of time, *P* < 0.001; interaction, *P* < 0.001) were evaluated every 3 days (A, B). Organs, such as heart, lungs, spleen, and livers were dissected at day 21, and organ weights were determined (C). (D‐F) BALB/c mice were injected subcutaneously with CT26 cells. Then, after 6 days of injection, mice were orally administrated with GRd (10 mg/kg body weight/day) or vehicle daily for 16 days. Mice without tumour inoculation was used as control health. Control (*n* = 6), CT26 + vehicle (n = 6), and CT26 + GRd 10 mg/kg (n = 6). Body weights (the main effect of treatment, *P* = 0.0464; the main effect of time, *P* < 0.001; interaction, *P* = 0.0003) and tumour volumes (the main effect of treatment, *P* < 0.001; the main effect of time, *P* < 0.001; interaction, *P* < 0.001) were evaluated every 3 days (D, E). Organs (heart, lungs, spleen, and liver) were dissected after 21 days, and the weights were determined (F). One‐way (C, F) or two‐way (A‐B, D‐E) ANOVA followed by Tukey's multiple comparisons were used to compare between data (ns, not significant; ^$^ *P* ≤ 0.05, ^$$^ *P* ≤ 0.01, ^$$$^ *P* ≤ 0.001, control health vs. LLC1 or CT26 + vehicle; ^#^ *P* ≤ 0.05, ^##^ *P* ≤ 0.01, ^###^ *P* ≤ 0.001, control health vs. LLC1 or CT26 + GRd; ^&^ *P* ≤ 0.05, ^&&^ *P* ≤ 0.01, ^&&&^ *P* ≤ 0.001, LLC1 or CT26 + vehicle vs. LLC1 or CT26 + GRd).
Figure S4. GRd reduces ROS levels.
C2C12 myoblast were incubated for 4 h with TNF‐α (20 ng/mL), with/without GRd (100 nM) as indicated, and then analysed by flow cytometry to analyse ROS levels. Representative FACS profiles were shown, and the data were presented as mean ± SEM of four independent experiments. One‐way ANOVA followed by Tukey's multiple comparisons were used to compare between data (*P ≤ 0.05; **P ≤ 0.01).
Figure S5. A model of the molecular pathway of GRd against muscle wasting.
GRd binds to STAT3, resulting in the inhibition of STAT3 phosphorylation and localization to the nucleus. Inhibition of STAT3 signalling leads to the repression of several atrophy‐inducing molecules. GRd also has ROS scavenging activity, which leads to a reduction of ROS levels and protects mitochondrial integrity.

## References

[1] Furrer R , Handschin C . Muscle Wasting Diseases: Novel Targets and Treatments. Annu Rev Pharmacol Toxicol 2019;59:315–339. 30148697 10.1146/annurev-pharmtox-010818-021041 PMC6701981

[2] Cruz‐Jentoft AJ , Sayer AA . Sarcopenia. Lancet 2019;393:2636–2646. 31171417 10.1016/S0140-6736(19)31138-9

[3] McGovern J , Dolan RD , Skipworth RJ , Laird BJ , McMillan DC . Cancer cachexia: a nutritional or a systemic inflammatory syndrome? Br J Cancer 2022;127:379–382. 35523879 10.1038/s41416-022-01826-2 PMC9073809

[4] Ma JF , Sanchez BJ , Hall DT , Tremblay AK , Di Marco S , Gallouzi IE . STAT3 promotes IFNγ/TNFα‐induced muscle wasting in an NF‐κB‐dependent and IL‐6‐independent manner. EMBO Mol Med 2017;9:622–637. 28264935 10.15252/emmm.201607052 PMC5412921

[5] Anker MS , Holcomb R , Muscaritoli M , von Haehling S , Haverkamp W , Jatoi A , et al. Orphan disease status of cancer cachexia in the USA and in the European Union: a systematic review. J Cachexia Sarcopenia Muscle 2019;10:22–34. 30920776 10.1002/jcsm.12402 PMC6438416

[6] Vainshtein A , Sandri M . Signaling Pathways That Control Muscle Mass. Int J Mol Sci 2020;21:4759. 32635462 10.3390/ijms21134759 PMC7369702

[7] Ham DJ , Börsch A , Lin S , Thürkauf M , Weihrauch M , Reinhard JR , et al. The neuromuscular junction is a focal point of mTORC1 signaling in sarcopenia. Nat Commun 2020;11:4510. 32908143 10.1038/s41467-020-18140-1 PMC7481251

[8] Sartori R , Romanello V , Sandri M . Mechanisms of muscle atrophy and hypertrophy: implications in health and disease. Nat Commun 2021;12:330. 33436614 10.1038/s41467-020-20123-1 PMC7803748

[9] Dumitru A , Radu BM , Radu M , Cretoiu SM . Muscle Changes During Atrophy. Adv Exp Med Biol 2018;1088:73–92. 30390248 10.1007/978-981-13-1435-3_4

[10] Kato H , Watanabe H , Imafuku T , Arimura N , Fujita I , Noguchi I , et al. Advanced oxidation protein products contribute to chronic kidney disease‐induced muscle atrophy by inducing oxidative stress via CD36/NADPH oxidase pathway. J Cachexia Sarcopenia Muscle 2021;12:1832–1847. 34599649 10.1002/jcsm.12786 PMC8718075

[11] Niu M , Song S , Su Z , Wei L , Li L , Pu W , et al. Inhibition of heat shock protein (HSP) 90 reverses signal transducer and activator of transcription (STAT) 3‐mediated muscle wasting in cancer cachexia mice. Br J Pharmacol 2021;178:4485–4500. 34265073 10.1111/bph.15625

[12] Fan M , Sun W , Gu X , Lu S , Shen Q , Liu X , et al. The critical role of STAT3 in biogenesis of tumor‐derived exosomes with potency of inducing cancer cachexia in vitro and in vivo. Oncogene 2022;41:1050–1062. 35034093 10.1038/s41388-021-02151-3

[13] Chen M , Xiao L , Dai G , Lu P , Zhang Y , Li Y , et al. Inhibition of JAK‐STAT Signaling Pathway Alleviates Age‐Related Phenotypes in Tendon Stem/Progenitor Cells. Front Cell Dev Biol 2021;9:650250. 33855026 10.3389/fcell.2021.650250 PMC8039155

[14] Powers SK , Ji LL , Kavazis AN , Jackson MJ . Reactive oxygen species: impact on skeletal muscle. Compr Physiol 2011;1:941–969. 23737208 10.1002/cphy.c100054 PMC3893116

[15] Sohn E‐H , Yang Y , Koo HJ , Park D , Kim Y‐J , Jang K , et al. Effects of Korean Ginseng and Wild Simulated Cultivation Ginseng for Muscle Strength and Endurance. Korean J Plant Res 2012;25:657–663.

[16] Phi LTH , Sari IN , Wijaya YT , Kim KS , Park K , Cho AE , et al. Ginsenoside Rd Inhibits the Metastasis of Colorectal Cancer via Epidermal Growth Factor Receptor Signaling Axis. IUBMB Life 2019;71:601–610. 30576064 10.1002/iub.1984

[17] Kim J‐K , Choi MS , Jeung W , Ra J , Yoo HH , Kim D‐H . Effects of gut microbiota on the pharmacokinetics of protopanaxadiol ginsenosides Rd, Rg3, F2, and compound K in healthy volunteers treated orally with red ginseng. J Ginseng Res 2020;44:611–618. 32617041 10.1016/j.jgr.2019.05.012 PMC7322745

[18] Wei W , Tweardy DJ , Zhang M , Zhang X , Landua J , Petrovic I , et al. STAT3 signaling is activated preferentially in tumor‐initiating cells in claudin‐low models of human breast cancer. Stem Cells 2014;32:2571–2582. 24891218 10.1002/stem.1752

[19] Park K , Ko YJ , Durai P , Pan CH . Machine learning‐based chemical binding similarity using evolutionary relationships of target genes. Nucleic Acids Res 2019;47:e128. 31504818 10.1093/nar/gkz743 PMC6846180

[20] Park JS , Ko K , Kim SH , Lee JK , Park JS , Park K , et al. Tropolone‐Bearing Sesquiterpenes from Juniperus chinensis: Structures, Photochemistry and Bioactivity. J Nat Prod 2021;84:2020–2027. 34236881 10.1021/acs.jnatprod.1c00321

[21] Yu W , Li C , Zhang W , Xia Y , Li S , Lin JY , et al. Discovery of an Orally Selective Inhibitor of Signal Transducer and Activator of Transcription 3 Using Advanced Multiple Ligand Simultaneous Docking. J Med Chem 2017;60:2718–2731. 28245116 10.1021/acs.jmedchem.6b01489

[22] Yu X , He L , Cao P , Yu Q . Eriocalyxin B Inhibits STAT3 Signaling by Covalently Targeting STAT3 and Blocking Phosphorylation and Activation of STAT3. PLoS ONE 2015;10:e0128406. 26010889 10.1371/journal.pone.0128406 PMC4444003

[23] Ma JF , Sanchez BJ , Hall DT , Tremblay AK , Di Marco S , Gallouzi IE . STAT3 promotes IFNgamma/TNFalpha‐induced muscle wasting in an NF‐kappaB‐dependent and IL‐6‐independent manner. EMBO Mol Med 2017;9:622–637. 28264935 10.15252/emmm.201607052 PMC5412921

[24] Chiappalupi S , Sorci G , Vukasinovic A , Salvadori L , Sagheddu R , Coletti D , et al. Targeting RAGE prevents muscle wasting and prolongs survival in cancer cachexia. J Cachexia Sarcopenia Muscle 2020;11:929–946. 32159297 10.1002/jcsm.12561 PMC7432590

[25] Silva KA , Dong J , Dong Y , Dong Y , Schor N , Tweardy DJ , et al. Inhibition of Stat3 activation suppresses caspase‐3 and the ubiquitin‐proteasome system, leading to preservation of muscle mass in cancer cachexia. J Biol Chem 2015;290:11177–11187. 25787076 10.1074/jbc.M115.641514 PMC4409274

[26] Yokoyama S , Ohno Y , Egawa T , Ohashi K , Ito R , Ortuste Quiroga HP , et al. MBNL1‐Associated Mitochondrial Dysfunction and Apoptosis in C2C12 Myotubes and Mouse Skeletal Muscle. Int J Mol Sci 2020;21:6376. 32887414 10.3390/ijms21176376 PMC7503908

[27] Eskiler GG , Bezdegumeli E , Ozman Z , Ozkan AD , Bilir C , Kucukakca BN , et al. IL‐6 mediated JAK/STAT3 signaling pathway in cancer patients with cachexia. Bratisl Lek Listy 2019;66:819–826. 31747761 10.4149/BLL_2019_136

[28] Pretto F , Ghilardi C , Moschetta M , Bassi A , Rovida A , Scarlato V , et al. Sunitinib prevents cachexia and prolongs survival of mice bearing renal cancer by restraining STAT3 and MuRF‐1 activation in muscle. Oncotarget 2015;6:3043–3054. 25460504 10.18632/oncotarget.2812 PMC4413636

[29] Zimmers TA , Fishel ML , Bonetto A . STAT3 in the systemic inflammation of cancer cachexia. Semin Cell Dev Biol 2016;54:28–41. 26860754 10.1016/j.semcdb.2016.02.009 PMC4867234

[30] Marceca GP , Londhe P , Calore F . Management of Cancer Cachexia: Attempting to Develop New Pharmacological Agents for New Effective Therapeutic Options. Front Oncol 2020;10:298. 32195193 10.3389/fonc.2020.00298 PMC7064558

[31] VanderVeen BN , Fix DK , Carson JA . Disrupted Skeletal Muscle Mitochondrial Dynamics, Mitophagy, and Biogenesis during Cancer Cachexia: A Role for Inflammation. Oxid Med Cell Longev 2017;2017:3292087. 28785374 10.1155/2017/3292087 PMC5530417

[32] Sriram S , Subramanian S , Sathiakumar D , Venkatesh R , Salerno MS , McFarlane CD , et al. Modulation of reactive oxygen species in skeletal muscle by myostatin is mediated through NF‐κB. Aging Cell 2011;10:931–948. 21771249 10.1111/j.1474-9726.2011.00734.x PMC5028794

[33] Tang K , Qin W , Wei R , Jiang Y , Fan L , Wang Z , et al. Ginsenoside Rd ameliorates high glucose‐induced retinal endothelial injury through AMPK‐STRT1 interdependence. Pharmacol Res 2022;179:106123. 35150861 10.1016/j.phrs.2022.106123

[34] Shen S , Liao Q , Liu J , Pan R , Lee SM‐Y , Lin L . Myricanol rescues dexamethasone‐induced muscle dysfunction via a sirtuin 1‐dependent mechanism. J Cachexia Sarcopenia Muscle 2019;10:429–444. 30793539 10.1002/jcsm.12393 PMC6463464

[35] Kim JH . Pharmacological and medical applications of Panax ginseng and ginsenosides: a review for use in cardiovascular diseases. J Ginseng Res 2018;42:264–269. 29983607 10.1016/j.jgr.2017.10.004 PMC6026386

[36] Kim YJ , Yamabe N , Choi P , Lee JW , Ham J , Kang KS . Efficient thermal deglycosylation of ginsenoside Rd and its contribution to the improved anticancer activity of ginseng. J Agric Food Chem 2013;61:9185–9191. 23984628 10.1021/jf402774d

[37] Wang P , du X , Xiong M , Cui J , Yang Q , Wang W , et al. Ginsenoside Rd attenuates breast cancer metastasis implicating derepressing microRNA‐18a‐regulated Smad2 expression. Sci Rep 2016;6:33709. 27641158 10.1038/srep33709 PMC5027393

[38] Go G‐Y , Jo A , Seo D‐W , Kim W‐Y , Kim YK , So E‐Y , et al. Ginsenoside Rb1 and Rb2 upregulate Akt/mTOR signaling–mediated muscular hypertrophy and myoblast differentiation. J Ginseng Res 2020;44:435–441. 32372865 10.1016/j.jgr.2019.01.007 PMC7195574

[39] Zhang G , Xia F , Zhang Y , Zhang X , Cao Y , Wang L , et al. Ginsenoside Rd Is Efficacious Against Acute Ischemic Stroke by Suppressing Microglial Proteasome‐Mediated Inflammation. Mol Neurobiol 2016;53:2529–2540. 26081140 10.1007/s12035-015-9261-8

[40] von Haehling S , Morley JE , Coats AJS , Anker SD . Ethical guidelines for publishing in the Journal of Cachexia, Sarcopenia and Muscle: update 2021. J Cachexia Sarcopenia Muscle 2021;12:2259–2261. 34904399 10.1002/jcsm.12899 PMC8718061

