# Supplementary material for: Ginsenoside Rd ameliorates muscle wasting by suppressing the signal transducer and activator of transcription 3 pathway
Source: J Cachexia Sarcopenia Muscle. 2022 Sep 20;13(6):3149–62. doi: 10.1002/jcsm.13084 (PMC9745546; doi:10.1002/jcsm.13084)
Supplement: Supplementary file 1 — Table S1. Predicted binding targets of GRd by ECBS Figure S1. Ginseng‐derived components protect from TNF‐α/IFN‐γ or conditioned media‐induced muscle cell atrophy. (A, B) Differentiated C2C12 myotubes were treated with various ginseng‐derived components in the presence or absence of TNF‐α (20 ng/mL) and IFN‐γ (100 U/mL) for 24 h and stained with anti‐MHC Ab. (A) Representative images were shown. (B) Average myotube diameter was measured by ImageJ software. (C, D) Differentiated C2C12 or HSKM myotubes were treated with conditioned media obtained from CT26, LLC1, or HT29, as indicated, for 24 h and stained with anti‐MHC Ab. (C) Representative images were shown. (D) Average myotube diameter was measured by ImageJ software. The data were shown as mean ± SEM of more than 100 myotubes from 10 randomly chosen fields. One‐way ANOVA followed by Tukey's multiple comparisons were used to compare between data (*P ≤ 0.05; **P ≤ 0.01; ***P ≤ 0.001). Figure S2. GRd maintained organ weights in an aged mouse model. (A) Body weights were monitored every 7 days. The main effect of treatment, P = 0.0249; the main effect of time, P < 0.001; interaction, P < 0.001. (B) Organs, such as heart, lungs, spleen, and livers were dissected at day 36. The weights of organs were determined. The data were shown as mean ± SEM. Two‐way (A) or one‐way (B) ANOVA followed by Tukey's multiple comparisons were used to compare between data. ns, not significant; $ P ≤ 0.05, $$ P ≤ 0.01, control adult vs. control old. # P ≤ 0.05, control adult vs. old+GRd; & P < 0.05, control old vs. old+GRd. Figure S3. GRd maintained body weights and organ weights in cachexia mouse models. (A‐C) C57BL/6 mice were injected subcutaneously (s.c.) with LLC1 cells. Then, after 6 days of injection, mice were orally administrated with GRd (10 mg/kg body weight/day) or vehicle daily for 16 days. Mice without tumour inoculation were used as control health. Control (n = 6), LLC1 + vehicle (n = 6), and LLC1 + GRd 10 mg/kg (n = 6). Bod [file JCSM-13-3149-s001.pdf]

Supplementary Table 1

Target(Uniprot)	Predicted Target Score	Protein	Gene
P19156	0.86719	Potassium-transporting ATPase alpha chain 1	ATP4A
P40763	0.81786	Signal transducer and activator of transcription 3	STAT3
Q3SZX4	0.80406	Carbonic anhydrase 3	CA3
Q96J66	0.77873	ATP-binding cassette sub-family C member 11	ABCC11
O15439	0.77453	Multidrug resistance-associated protein 4	ABCC4
P34976	0.76957	Type-1 angiotensin II receptor	AGTR1
P20648	0.76494	Potassium-transporting ATPase alpha chain 1	ATP4A
Q5T3U5	0.76338	Multidrug resistance-associated protein 7	ABCC10
P05024	0.75114	Sodium/potassium-transporting ATPase subunit alpha-1	ATP1A1
O95342	0.75104	Bile salt export pump	ABCB11
P42229	0.74479	Signal transducer and activator of transcription 5A	STAT5A
P14679	0.74009	Tyrosinase	TYR
P09626	0.72338	Potassium-transporting ATPase alpha chain 1	Atp4a
P05023	0.70577	Sodium/potassium-transporting ATPase subunit alpha-1	ATP1A1
P06685	0.70193	Sodium/potassium-transporting ATPase subunit alpha-1	Atp1a1
Q7KZA3	0.6856	Ferrochelatase	DKFZp686P18130
P22830	0.68489	Ferrochelatase, mitochondrial	FECH
O43193	0.68164	Motilin receptor	MLNR
P02768	0.68115	Serum albumin	ALB
P13637	0.67143	Sodium/potassium-transporting ATPase subunit alpha-3	ATP1A3

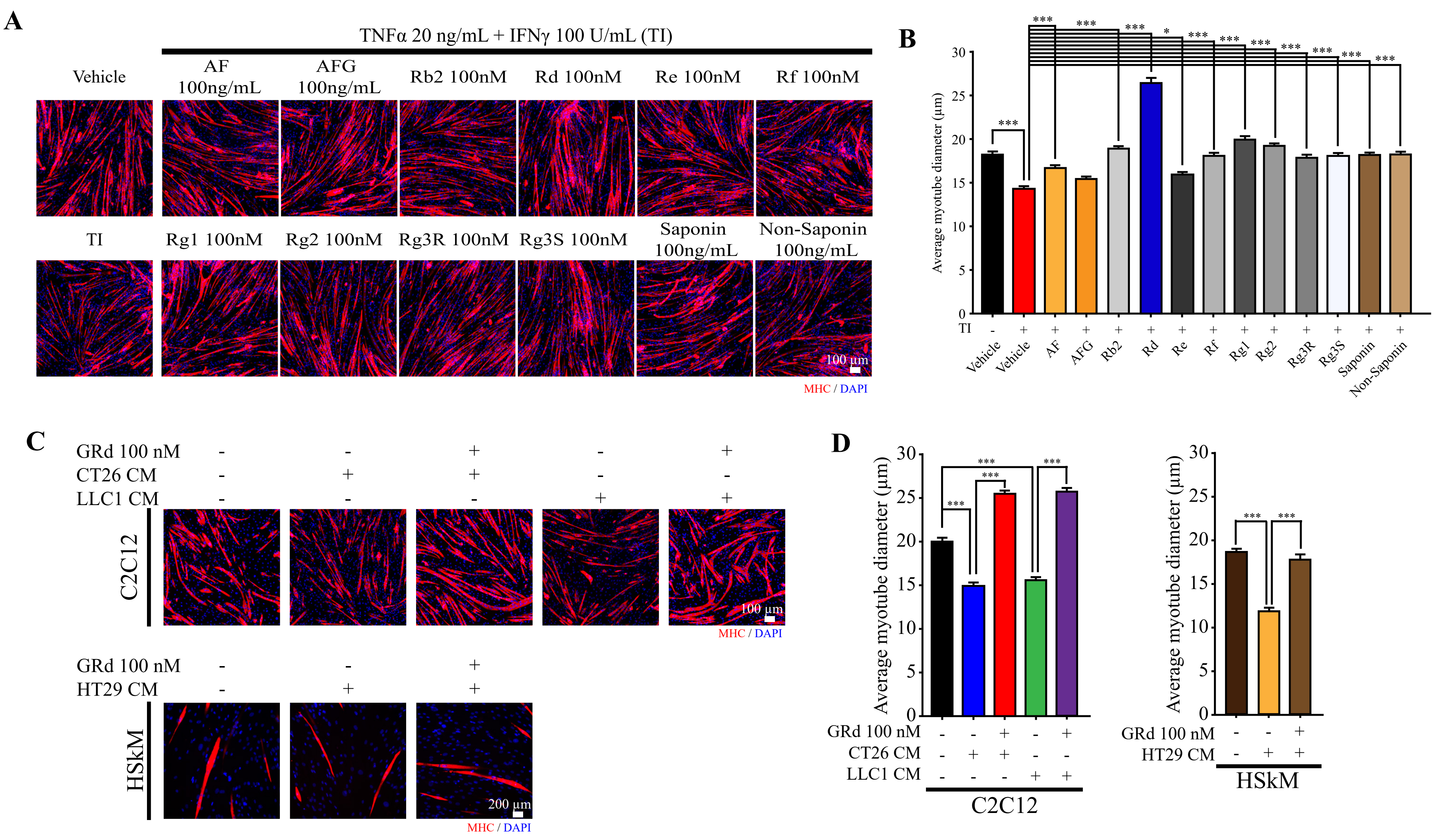

Supplementary Figure 1

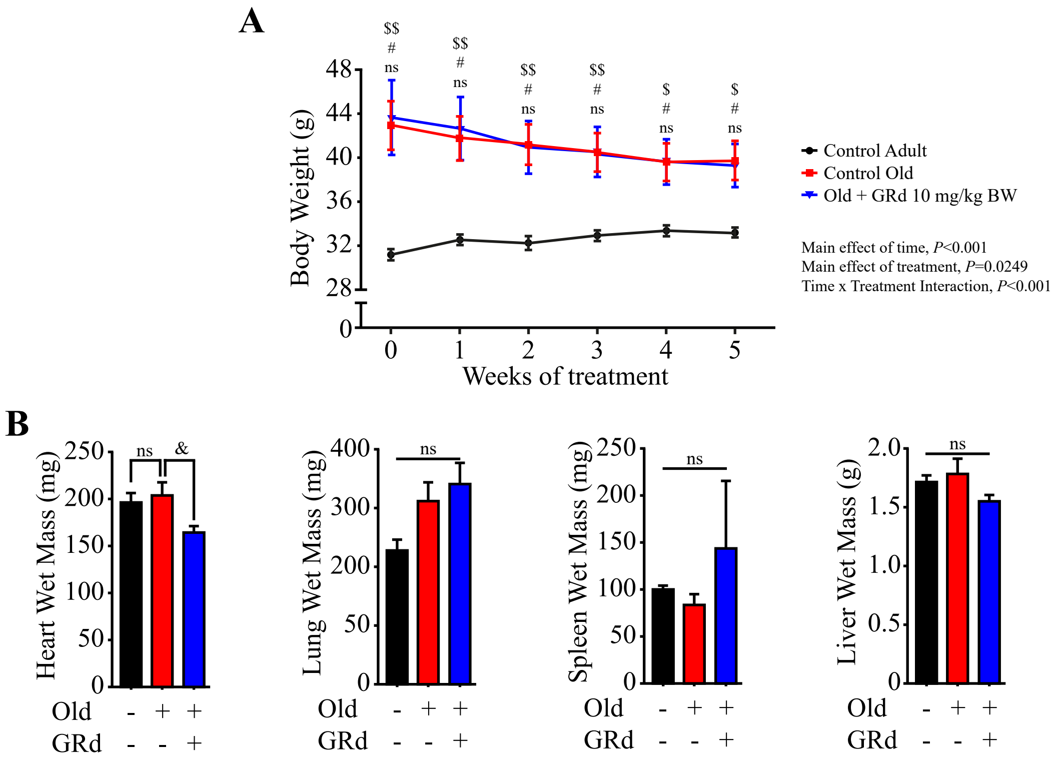

Supplementary Figure 2

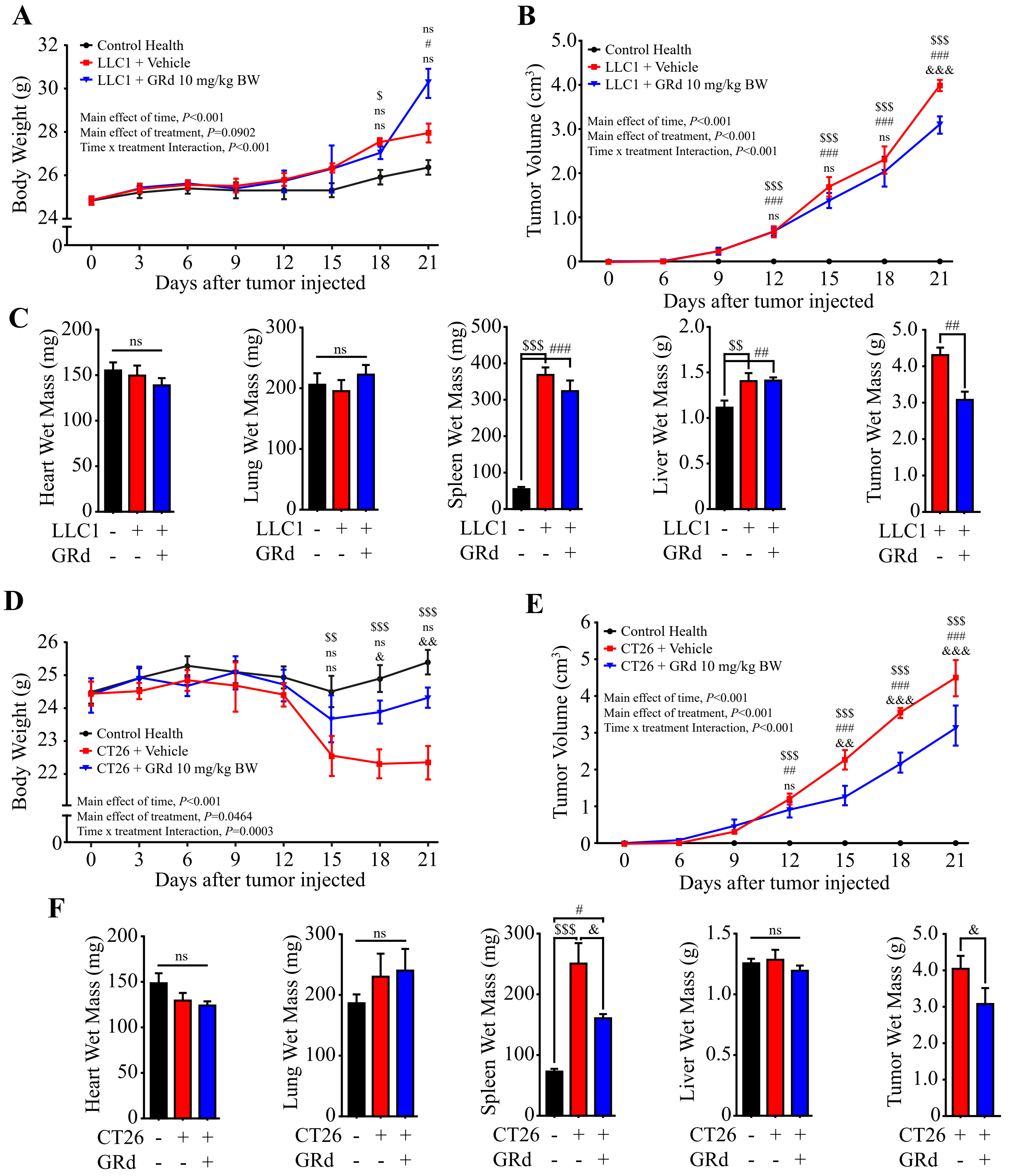

Supplementary Figure 3

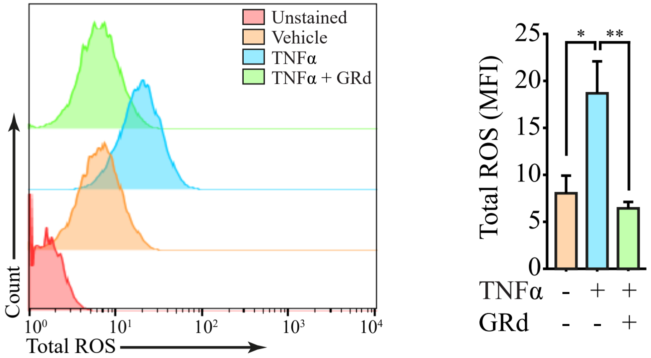

Supplementary Figure 4

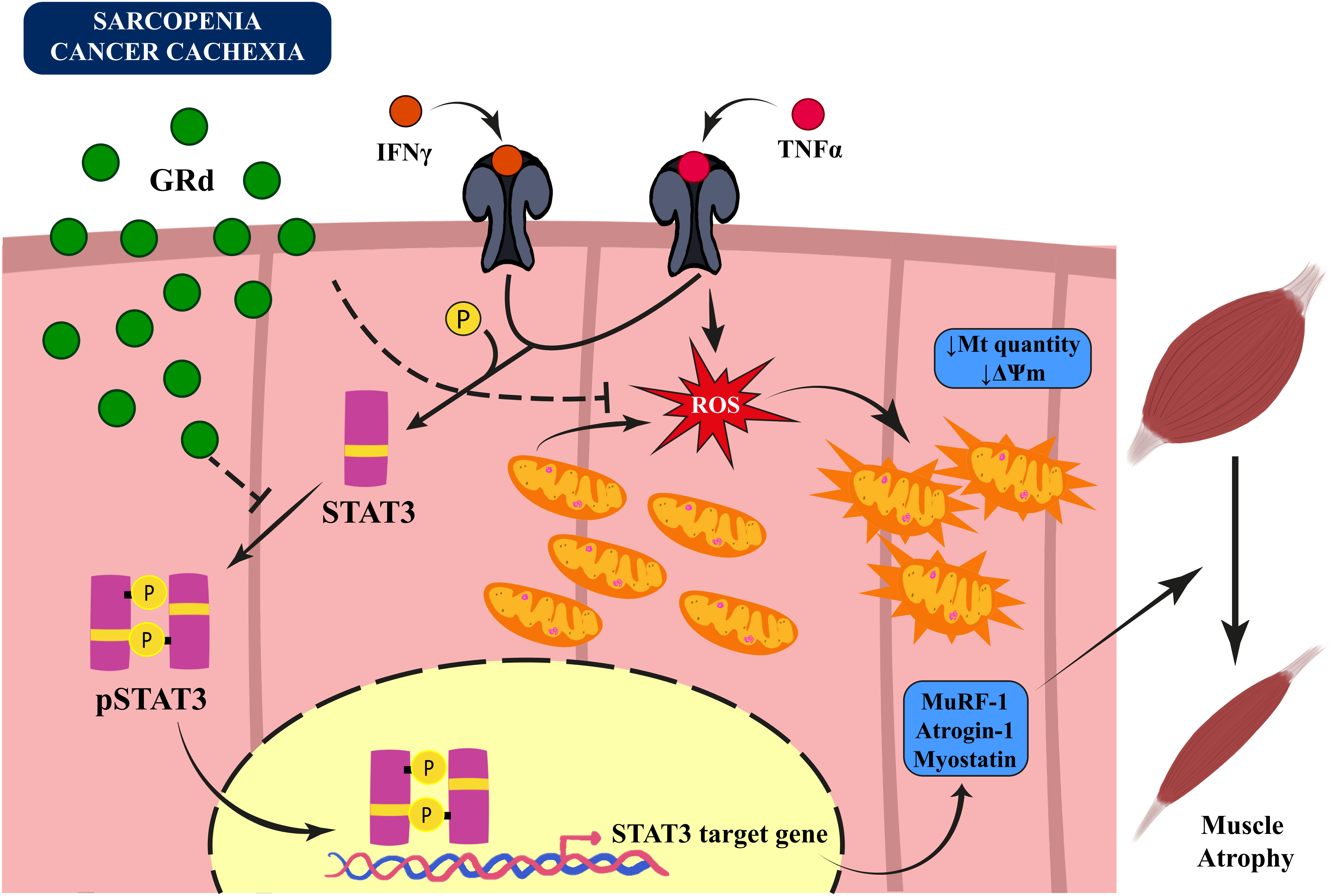

Supplementary Figure 5
